# Therapeutic potential of targeting mirnas to prostate cancer tumors: using psma as an active target

**DOI:** 10.1080/23723556.2022.2136476

**Published:** 2022-10-24

**Authors:** Amir Yarahmadi, Romoye Sohan, Brenna McAllister, Leslie A. Caromile

**Affiliations:** a Vascular and Endovascular Surgery Research Center, Mashhad University of Medical Sciences, Mashhad, Iran; b Center for Vascular Biology, University of Connecticut Health Center, Farmington, CT, USA

**Keywords:** PSMA, prostate cancer, miRNA, therapy, targeted delivery

## Abstract

Prostate cancer (PC) is a commonly diagnosed malignancy in men and is associated with high mortality rates. Current treatments for PC include surgery, chemotherapy, and radiation therapy. However, recent advances in targeted delivery systems have yielded promising new approaches to PC treatment. As PC epithelial cells express high levels of prostate-specific membrane antigen (PSMA) on the cell surface, new drug conjugates focused on PSMA targeting have been developed. microRNAs (miRNAs) are small noncoding RNAs that regulate posttranscriptional gene expression in cells and show excellent possibilities for use in developing new therapeutics for PC. PSMA-targeted therapies based on a miRNA payload and that selectively target PC cells enhances therapeutic efficacy without eliciting damage to normal surrounding tissue. This review discusses the rationale for utilizing miRNAs to target PSMA, revealing their potential in therapeutic approaches to PC treatment. Different delivery systems for miRNAs and challenges to miRNA therapy are also explored.

## Introduction

Prostate cancer (PC) is one of the leading causes of mortality in male cancer patients worldwide.^1^ Effective PC management remains an ongoing challenge, with androgen deprivation therapy (ADT) the current standard PC treatment.^2^ Although this course of treatment improves the survival and quality of life for individuals with PC, most men eventually progress to metastatic castration-resistant PC (mCRPC).^3^ Advanced PC (including castration-sensitive and castration-resistant disease) is commonly managed with androgen axis – targeted therapies, such as abiraterone acetate and enzalutamide, docetaxel-based chemotherapy, or radium Ra-223 dichloride.^4^ Unfortunately, only approximately 50% of patients with advanced disease respond favorably to these therapies, with the other 50% developing resistance and exhibiting survival rates of only 5% − 30%.^2^ There are many reasons for treatment failure: Factors such as a mutation in the androgen receptor (AR) drug-binding domain, tumor heterogeneity, and vascular permeability all negatively affect efficient drug delivery to tumor sites. Moreover, many currently used drugs exhibit limited specificity and often produce deleterious effects on healthy peripheral tissues.^5,6^ Therefore, targeted drug delivery holds immense potential to improve cancer treatment by selectively providing effective therapies at tumor sites. Ideally, these therapies not only specifically recognize tumors but also target survival pathways that the tumor has leveraged to achieve drug resistance.

PC cells within prostate tumors express many tumor-associated antigens that can be potentially targeted for cancer diagnosis, treatment, and selective drug delivery.^7,8^ Prostate-specific membrane antigen (PSMA), a type II transmembrane protein found predominantly on the surface of prostate epithelial cells, is among these,,^9–11^ PSMA is expressed on the epithelium of nearly all PCs, and its increased expression correlates with progression to castration resistance and metastatic disease.^12–14^ The *cytoplasmic domain* of PSMA contains a motif that signals the internalization of PSMA via clathrin-coated pits,^15,16^ and clinical technologies utilize this pathway to enhance the delivery of radiopharmaceuticals into the tumor, with ^17^Ga-PSMA-11 PET/CT and ^177^Lu-PSMA-617^18–20^ leading the way. Studies with both antibody-drug conjugates (ADCs) and small-molecule drug conjugates (SMDCs) have demonstrated encouraging results.^21–27^ thus highlighting the continued interest in PSMA in biomedical, translational medicine, and pharmaceutical fields.^28^

microRNAs (miRNAs) are conserved 21–25-nucleotide-long noncoding molecules that play essential roles in regulating gene expression and participate in various biological processes,^29^ including roles in cancer ^30,31^ by functioning as a tumor suppressor ^32^ or as an onco-miRNA that represses the expression tumor suppressor genes such as p53.^32^ Each miRNA has the potential to target many genes. By using a single microRNA to silence multiple genes, several signaling pathways can be simultaneously regulated, which may minimize compensatory mechanisms that cause therapeutic resistance. Therefore, manipulating cellular miRNA levels with modified oligonucleotides that mimic or inhibit miRNA function has led to the extensive research and development of miRNAs as therapeutics.^33^ Loss-of-function approaches have led to superior research results, as they reveal processes dependent on physiological miRNA levels; in contrast, exogenous miRNA added to the system can lead to repressed activity of targeted mRNAs in nonphysiological contexts since miRNA – target interaction is highly concentration dependent. The expression of many miRNAs is tissue-specific and altered in different diseases, including PC. These alterations can significantly affect tumor cell growth and survival.^34,35^ Furthermore, miRNAs are considered valuable diagnostic biomarkers and potential therapeutic targets in cancer;^36^ for example, miRNA-15a, miRNA-21, miRNA-34a, miRNA-153, and miRNA-17 have been connected with PC pathogenesis.^37,38^ Therefore, miRNAs that perturb human disease pathways are potentially powerful candidates for therapeutic intervention against various pathological conditions, including PC.

Although the understanding of miRNA biology has grown exponentially since its discovery in 1993 by Lee and colleagues,^39^ a more comprehensive assessment of the strengths and limitations of miRNA-based approached for PC therapy is still necessary. In this review, we discuss potential therapeutic strategies of targeting PSMA to deliver specific miRNA payloads exclusively to PC tumors as well as provide insight into various delivery systems for miRNAs and the challenges to using these systems for therapy.

## miRNA biogenesis and mechanisms of action

**Figure 1.**
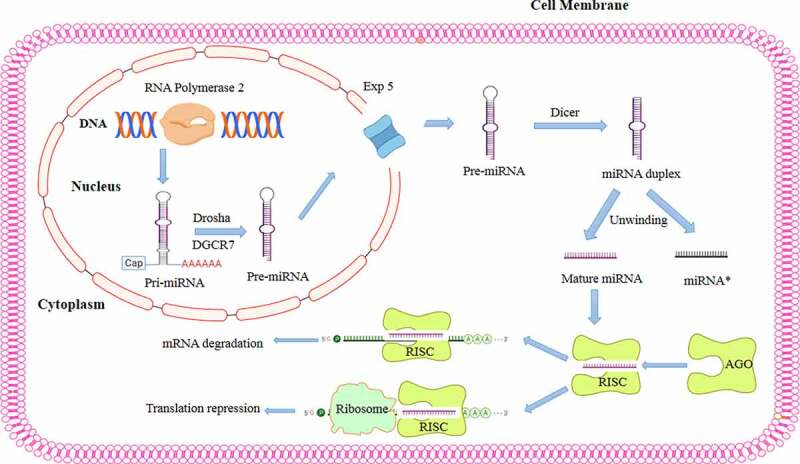
miRNA biogenesis and mechanisms of action in posttranscriptional gene regulation. miRNA biogenesis begins with miRNA transcription from DNA via the action of RNA polymerase II to generate primary hairpin miRNA (pri-miRNA). Then, pri-miRNA is cleaved by the RNase III drosha and its binding partner DiGeorge syndrome critical region gene 8 (DGCR8), which recognizes the hairpin structures in pri-miRNA and processes them to form precursor miRNA (pre-miRNA). The resulting pre-miRNA is exported to the cytoplasm by Exportin-5, a Ran-GTP-dependent dsRNA-binding protein. In the cytoplasm, another RNase III enzyme, dicer, further processes pre-miRNA, cleaving its hairpin and thus producing a mature miRNA duplex. Then, one strand is loaded into an argonaute (AGO) family member to form the miRNA-induced silencing complex (RISC) that recognizes the mRNA target via sequence complementarity, resulting in mRNA degradation or translation inhibition.

miRNA biogenesis is a complex process that begins with nuclear transcription mediated by RNA polymerase II forming a primary transcript known as primary miRNA (pri-miRNA).^40^ Pri-miRNAs contain a unique hairpin stem‒loop structure and a single-stranded sequence of differing lengths that can potentially harbor hundreds of kilobases.^41^ The nuclear complex contains the ribonuclease III enzyme Drosha as well as a double-stranded RNA (dsRNA)-binding protein (DiGeorge syndrome critical region 8 protein (DGCR8)), which facilitate Drosha removal of approximately 11 bp from each side of the hairpin stem of a pri-miRNA, resulting in precursor miRNA (pre-miRNA).^41,42^ A pre-miRNA is an approximately 70-nucleotide stem‒loop structure that is transported from the nucleus to the cytoplasm by exportin-5 (×PO5), a Ran-GTP-dependent dsRNA-binding protein.^43^ Once in the cytoplasm, the pre-miRNA is processed by the ribonuclease III enzyme Dicer to form a mature, 22-nucleotide miRNA duplex.^44^ The mature miRNA is then incorporated into the miRNA-induced silencing complex (RISC),^45,46^ through which it regulates gene expression through translational repression mediated by mRNA deregulation (Figure 1).^45^

## miRnas in PC progression

miRNAs play crucial roles in critical cellular processes such as cell proliferation, differentiation, cell cycle progression, apoptosis, angiogenesis, the epithelial-mesenchymal transition (EMT), and metastasis during cancer progression.^47^ Hao et al. showed that miRNA-101 inhibited PC cell proliferation by inhibiting cyclooxygenase-2 (COX-2) gene expression, inhibiting the activation of the COX-2/PGE2/EGFR pathway, which mediates cell proliferation during inflammation.^48^ COX-2 is an inducible isozyme of COX, a key enzyme in converting arachidonic acid to prostaglandins and other eicosanoids. COX-2 is highly expressed in several human cancers and cancer cell lines, including PC tumor cells, and activates the PGE2/EGFR pathway, leading to cell proliferation via extracellular signal-regulated kinase 2 (ERK2) activation.^49,50^ A study by Zhu et al. demonstrated that miRNA-136 suppressed PC cell proliferation and invasion by targeting mitogen-activated protein kinase 4 (MAP2K4a) *in vitro* ^*51*^. MAP2K4a can increase androgen receptor expression/activation and promote PC tumor progression via noncanonical activation of AKT.^51^ Moreover, Wang et al. showed that miRNA-182 upregulation increased the expression of important regulators of cell cycle progression, namely c-MYC and cyclin D1, leading to uncontrolled proliferation of the LNCaP and PC3 immortalized human PC cell lines.^52^ As miRNAs control the expression of cell cycle-related genes, identifying critical miRNAs involved in the cell cycle can lead to better treatment opportunities for cancers, including PC.^53^ For instance, miRNA-193a functions as a tumor suppressor, and its expression is lower in PC tissues compared to that in benign prostatic hyperplasia.^54^ In addition, Liu et al. demonstrated that miRNA-193a overexpression inhibited cell growth by targeting cyclin D1 and promoting G1-phase cell cycle arrest in the DU-145 immortalized human PC cell line as well as in PC3 cells.

Apoptosis is a complex process that involves many signaling pathways that can be modulated by miRNAs. Ma et al. showed that miRNA-143 decreases the proliferation and induces the apoptosis of LNCaP cells by suppressing the expression of the integral outer mitochondrial membrane protein BCL2, which inhibits cell death.^55^ A study by Ostadrahimi et al. demonstrated that miRNA-185, miRNA-30c, and miRNA-1266 were downregulated in PC tissues compared to healthy control tissues,^56^ resulting in antiapoptotic BCL2 and BCL2-XL gene upregulation and a reduced apoptosis rate.^56^

**Figure 2.**
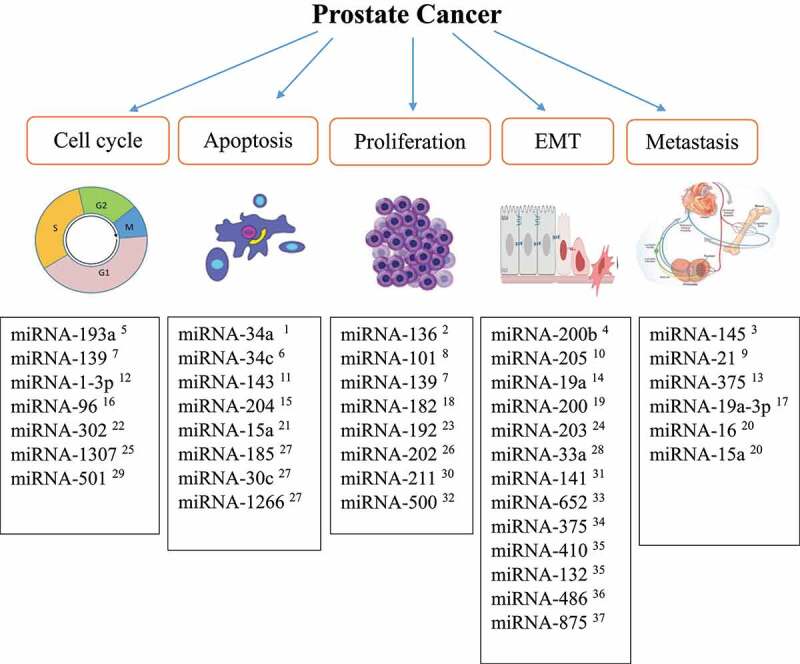
Summary of the important miRnas involved in PC.

Among the crucial outcomes of cancer cell progression to metastatic phenotype acquisition is the EMT.^57^ Several miRNAs have been suggested to regulate the expression of genes involved in the EMT, and a reduction in their expression leads to cancer invasion and metastasis.^58^ For example, miRNA-200b targets the zinc-finger E-box-binding homeobox 1 and 2 genes (ZEB1 and ZEB2), Bim1, and E-cadherin. ZEB1/ZEB2 directly bind to the E-box in the promoter of the adhesion molecule E-cadherin, recruiting transcriptional corepressors and inducing the EMT in PC.^59–62^ miRNA-200b action is crucial for cells to maintain their epithelial phenotype and prevent the EMT and tumor metastasis.^63^ Yu et al. showed that administration of miRNA-200b downregulated the expression of ZEB1 and ZEB2 in PC3 cells and reversed the EMT, attenuating EMT phenotype acquisition.^63^ A study by Gandellini et al. demonstrated that miRNA-205 plays a vital role in the EMT by targeting integrin-β4, laminin, and matrix metalloproteinase 2 (MMP2), which are necessary for interactions between the PC cell cytoskeleton and the extracellular matrix.^17,64,65^ A decrease or loss in miRNA-205 expression results in increased cell proliferation and invasion and changes to prostate cell characteristics, moving them toward a mesenchymal phenotype.^66^ The essential miRNAs involved in PC pathogenesis are summarized in Figure 2.

## Targeted systems for miRNA delivery

Primary PC results in a highly vascular tumor derived from proliferating prostatic epithelial cells.^67^ Tumor vascularity significantly affects tumor growth and drug responsiveness because it influences tumor blood flow, oxygenation, and the permeability of chemotherapeutic drugs into the tumor.^68,69^ Successfully targeted drug delivery systems are small (from 10 to 100 nm in diameter), remain stable in the circulation, accumulate in leaky tumor vasculature via the enhanced permeability and retention (EPR) effect, and enable targeted delivery of specific-ligand-modified drugs and drug carriers to areas with limited access.^70,71^ Targeting specific cancer cells is a crucial characteristic of drug delivery systems as targeting enhances therapeutic efficacy without eliciting damage to normal surrounding tissue or causing a bystander effect.^72^ Recent advances in drug delivery systems have suggested promising miRNA-based approaches for the delivery and treatment of different diseases, including PC.^73^ These advances can be broadly classified into two categories: passive and active targeting approaches.

### Passive targeting

Passive targeting exploits the biological characteristics of tumorous and normal tissue to deliver a drug to the target site, where it can exert a therapeutic effect. Tumor growth and metastasis depend on angiogenesis to provide an adequate supply of oxygen and nutrients to the tumor and to remove waste products.^74^ However, this new tumor vasculature is often defective and leaky, hindering the delivery and effectiveness of systemically administered therapeutic cancer drugs to the tumor.^75^

Many limitations have prevented miRNAs from becoming optimal candidates for this type of delivery, including the stability of the miRNA in the circulation, its ability to accumulate in pathological sites with differing vascular permeability and nonspecific distribution, and most importantly, the fact that one miRNA has the potential to target many different mRNAs leading to a nonspecific effect.^76,77^ These challenges to miRNA usage for targeted drug delivery and some possible solutions are highlighted in Table 1.

**Table 1.** Challenges to miRNA usage for targeted drug delivery and possible solutions.

Challenges	Possible Solutions
Degradation of miRNAs by nucleases	Changing the surface charge and improving their stability
Filtration in the spleen and kidneys Destruction by macrophages while in the circulatory system	Chemical modification and/or local administration
Penetration through the cell membrane and extracellular matrix	Active targeting via specific ligands and use of cell-penetrating moieties
Poor endosomal release and intracellular localization problems	Use of conjugating peptides, lytic reactions, or miRNA sponges
Ability to target multiple mRNAs	Local administration and active targeting via specific ligands

### Active targeting

Active targeting affects cancer cells through direct interactions between ligands and target molecules that are overly abundant on the surface of cancer cells, allowing the carriers to distinguish targeted cells from healthy cells.^78^ The drug carriers are internalized into the cell via receptor-mediated endocytosis, and then, the payload is released.^79^ This active ligand-specific targeting is particularly suitable for miRNA-mediated drug delivery applications. The most common active targeting carriers for miRNAs are generated from peptides, antibodies, aptamers, and nanoparticles, which help miRNAs specifically target tumor cells.^80^ In summary, active targeting is a precise mechanism for targeting tumor cells that reduces the need for a high number of miRNAs, which is required for passive targeting, and thus prevents unwanted side effects.^81^

PSMA is overly abundant on the surface of PC epithelial cells and thus has been used as a successful target for PC management.^82^ Interestingly, PSMA is expressed on the surface of endothelial cells in the tumor neovasculature in many other types of cancers, including breast, lung, gastric, colorectal, pancreatic and renal cell carcinoma, and bladder cancers. Therefore, using PSMA to carry therapeutic miRNA payloads may be broadly applicable to cancers in addition to PC.^83^ We discuss the most common active targeting PSMA-based carriers used for miRNA in PC.

### PSMA-targeting peptides and proteins as miRNA carriers

Peptide- and protein-based carriers have been broadly used for miRNA delivery because of the ability of the positive charged amino acids to interact with negatively charged nucleotides.^84^ For instance, Jin et al. developed a novel combinatorial phage biopanning procedure to identify PSMA-specific-targeting peptides as carriers for targeted drug delivery to PC cells.^85^ They reported that a novel PSMA-specific-targeting peptide named GTI, on the basis of its amino acid sequence, exhibited high binding affinity and selectivity for PSMA and PSMA-positive PC cells. Specifically, GTI mediated the internalization of the apoptotic KLA peptide into PSMA-positive LNCaP cells and induced cell death. Moreover, FAM-labeled GTI displayed high and specific tumor uptake in nude mice bearing human PC xenografts. It can be employed as a PSMA-specific ligand.^85^ Although this system may be an excellent tool for PC diagnosis and targeted drug delivery to PC, to date, no study on the use of this system for miRNA delivery via PSMA targeting in PC has been reported.

### Anti-PSMA antibodies as miRNA carriers

Antibody-based approaches have been widely used to target tumor cells via active targeting with specific drug carriers in cancer.^86^ Henry et al. used MLN2704, an antibody-chemotherapeutic conjugate consisting of a monoclonal antibody specific to PSMA conjugated to the drug maytansinoid 1 (DM1), which has microtubule-depolymerizing activity. After MLN2704 binds to PSMA through its specific antibody, MLN2704-PSMA is internalized, and DM1 is released into the cells, leading to cancer cell death. The Henry et al. study demonstrated that MLN2704 showed antitumor activity in an animal model of PC, whereas an unconjugated antibody showed no antitumor activity and DM1 alone showed weak tumor-suppressing activity *in vivo* .^87^ Rege et al. focused on designing and generating an amphipathic fusion peptide to destroy PC cells. Amphipathic lytic peptides exert cytotoxic effects on PC cells via depolarization of mitochondrial membranes and the induction of apoptosis.^88^ This group used PSMA-targeted peptides and antibodies against PSMA to precisely deliver cytotoxic amphipathic lytic peptides to PSMA-positive LNCaP cells. The results showed that, compared to the peptides, the antibodies more efficiently targeted the PC cells. Additionally, the group compared the cytotoxic activity of fusion peptides and antibody conjugates and found that treatment with fusion peptides induced oncotic/necrotic death in LNCaP cells; moreover, treatment with the antibody conjugates caused apoptotic death in these cells.^88^ Several anti-PSMA monoclonal antibodies with cytotoxic agents have been introduced for radioimmunotherapy application to target PSMA-expressing cells.^89^ For example, Behe et al. used the ^177^Lu-labeled anti-PSMA monoclonal antibody 3F11 to target PC cells in a mouse xenograft model. Their results indicated that ^177^Lu-labeled anti-PSMA 3F11 showed high specificity and affinity for a xenograft mouse model, making it a potential candidate for radioimmunotherapeutic applications in PC.^90^ However, the literature on the use of this system for miRNA delivery in PC treatment is rare.

### PSMA-directed aptamers as miRNA carriers

Aptamers are short single-stranded DNA or RNA oligonucleotides with a unique three-dimensional structure that enables its selective binding to specific receptors or protein targets,^91^ making them excellent drug delivery platforms.^92^ Conjugation of aptamers to miRNAs is a new method to deliver miRNAs precisely to PC cells.^93^ Dassie et al. developed an RNA aptamer (A9 g) that selectively inhibited PSMA enzyme activity and functioned as a smart drug for PC treatment. Because PSMA activity plays a crucial role in PC progression, this group showed that PC tumor treatment with the A9 g aptamer in a murine model significantly reduced cell migration and invasion *in vitro* and metastasis to bone *in vivo*.^94^ Wu et al. showed that targeting PC with miRNA-15a and miRNA-16-1 (potent tumor suppressors in PC) through the RNA aptamer A10–3.2, which specifically targets PC cells with PSMA residing on their surface, was beneficial for the selective killing of PC cells *in vitro*.^95^ Another study by Ye et al. revealed that aptamers in a compound with hyperbranched polyamidoamine (HPAA) and polyethylene glycol (PEG) and used for targeting PSMA-positive LNCaP cells via miRNA-133a-3p delivery facilitated miRNA-133a-3p delivery into LNCaP cells and showed excellent cytotoxicity in these cells. Furthermore, in an *in vivo* mouse model of PC, systemic injection of the APT-HPAA-PEG/miRNA-133a-3p compound inhibited tumor growth and prolonged animal survival.^96^

### PSMA nanoparticles as miRNA carriers

Nanoparticles (NPs) are essential carriers in cancer prevention and therapy because they can be generated with unique sizes and shapes that enable them to deliver miRNAs and other chemotherapeutic agents.^97^ Silica, gold, and iron oxide NPs have been primarily used for miRNA delivery in cancer treatment.^98^ Luo et al. conjugated a PSMA-targeting ligand named PSMA-1 to gold NPs (AuNPs) and found that these PSMA-1-AuNPs showed greater uptake by PSMA-expressing PC3 cells compared to cells lacking PSMA receptors. As gold can increase radiotherapy sensitization, significantly enhanced radiotherapy efficacy was observed with these PSMA-targeting AuNPs.^99^ Additionally Binzel et al. reported that NPs containing an anti-PSMA RNA aptamer as the targeting ligand and carrying anti-miRNA-17 or anti-miRNA-21 (two common oncogenes) suppressed miRNA oncogenic activity in PC, showing significant knockdown of miRNA-17 and miRNA-21 and upregulation of phosphatase and tensin homolog (PTEN), a negative regulator of tumor growth in both *in vitro* and *in vivo* models of PC.^100^ Saniee et al. developed a docetaxel-loaded NP consisting of poly(lactic-co-glycolic acid) polyethylene glycol (PLGA-PEG) conjugated with a urea-based anti-PSMA ligand named glutamate-urea-lysine (Glu-urea-Lys) to deliver docetaxel for PC treatment. The uptake of these NPs by PSMA-positive LNCaP and PSMA-negative PC3 cells was analyzed. The results showed that docetaxel uptake was more efficient in the PSMA-positive cells when compared to the control. In addition, this group showed that compared to that of PSMA-targeted NP-carried drugs, the toxicity of untargeted NP-carried drugs was reduced by more than 70%. Finally, the NPs specifically targeting PSMA-positive PC cells showed enhanced the antitumor efficacy mediated via docetaxel.^101^

## Challenges to miRNA therapy

Similar to other treatment strategies, challenges and limitations have been identified in using miRNAs in cancer treatment.^102^ The first three challenges to miRNA therapy are caused by 1) nucleases quickly degrading naked miRNAs in the circulatory system,^103^ 2) miRNAs quickly cleared through the kidney,^102^ and 3) naked miRNAs frequently inducing immune responses and being eliminated from the circulation by macrophages, thus requiring high-dose administration, which subsequently leads to toxicity.^104,105^ To address these three challenges, several different approaches have been employed to alter the miRNA surface charge through structural chemical modifications:^106^ 1) locked nucleic acid (LNA) modification, 2) ribose 2′-OH group modification, 3) peptide nucleic acid (PNA) modification, and 4) backbone modification.^106^

LNA antisense oligonucleotides have been the most extensively studied miRNA structural modifications and have been demonstrated to enhance endonuclease resistance and increase biodistribution and to exhibit a lower toxicity profile than unmodified miRNA.^107^ The most common groups used to as a substitution for 2′-OH are 2′-O-methyl, 2′-O-methoxyethyl, and 2′-O-fluoro groups. Substitution with these chemical groups enhanced stability, increased binding affinity, and increased the effectiveness of miRNA inhibition *in vivo*.^108^ PNAs are uncharged oligonucleotide analogs. A phosphodiester backbone replacement with PNAs produces *N*-(2-aminoethyl)-glycine units. PNA recognizes single-stranded nucleic acids with extremely high affinity and sequence selectivity. Although uncharged, PNAs increase oligonucleotide stability, making them suitable for therapeutic approaches.^109^ Backbone modification is another strategy in which one of the critical atoms in an oligonucleotide is replaced to create a more stable oligonucleotide with therapeutic applications. The most widely used backbone-modified oligonucleotides are generated by replacing one oxygen atom with a sulfur atom. This modification has been shown to enhance nuclease resistance. However, modified oligonucleotides exhibit a short circulation half-life and low binding affinity.^110^

The fourth challenge in miRNA therapy is low penetration through the cell membrane and the ECM. miRNAs are hydrophilic and therefore cannot cross cell membranes, despite their negative charge.^111^ Different approaches to help miRNAs cross the cell membrane include active targeting via peptides and conjugation with lipid-soluble compounds such as cholesterol.^112^ For example, Fabani et al. designed anti-miRNA-122 conjugated to penetratin, a cell-penetrating peptide (also known as a protein transduction domain), enabling delivery of miRNAs through the cell membrane *in vitro*.^113^

The fifth challenge in miRNA therapy is endosomal escape and intracellular localization.^114^ To enhance endosomal release, conjugating peptides and probes have been developed. For example, Xie et al. used chloroquine-containing 2-(dimethylamino)ethyl methacrylate (DMAEMA) copolymers to enhance miRNA delivery by increasing the endosomal escape rate. Their results showed that miRNA delivery efficiency was increased by using chloroquine-DMAEMA copolymers in breast cancer cells.^115^

The sixth challenge involves miRNA targeting of multiple mRNAs and the subsequent toxicity caused by off-target gene silencing. miRNAs inhibit the expression of target genes by imperfect base pairing with target mRNA, allowing a single miRNA to regulate the expression of multiple genes, acting as a potent multidrug. For example, the tumor-suppressing miRNA miR-34 can downregulate genes involved in cell proliferation (c-MYC, androgen receptor), angiogenesis (VEGF), apoptosis inhibition (BCL2), and the immune response (PD-L1), resulting in a potent antitumor response.^116,117^ Localized use of miRNAs and active targeting delivery systems can reduce off-target gene silencing and the number of possible side effects.^118^ Furthermore, miRNAs may face obstacles in reaching their targets due to competitors that interfere with their functions. Increasing evidence suggests that competitive endogenous RNAs (ceRNAs) can prevent the downregulation of mRNA targets by binding through their own miRNA-binding sites.^119^ These ceRNAs need to be blocked before miRNA targeted therapy can exert the maximum effect. We present a summary of the most critical challenges to miRNA therapy and potential solutions to overcome these challenges through new drug delivery technologies in Table 1.

## Conclusions and future perspectives

During the past decade, advancements in studies on miRNA functions and their essential roles in cancer have led to many possibilities for miRNA therapeutic applications. To date, many different strategies have been studied to find a suitable method for miRNA delivery in cancer treatment.^120^ Nevertheless, the specific properties of miRNAs and various physiological obstacles remain the main limitations of *in vivo* miRNA delivery.^121^ However, new opportunities and discoveries being presented by targeted miRNA research are increasing the possibility of using miRNA therapy for cancer treatment.^122^ In the attempts to use miRNA in cancer treatment, PSMA is being tested as a reliable and specific target for developing a targeted delivery system with potential application in PC, one of the most common cancers worldwide.^95,96^ As targeted miRNA therapy systems mediated through PSMA may increase the efficacy and prevent toxic effects on other human cells, new treatment strategies for using PSMA in PC treatment will be valuable for further studies. Furthermore, future works are needed for designing and optimizing an effective delivery system for miRNAs targeting PSMA and potential combinations of these therapies, along with other therapeutic strategies, for the long-term treatment of PC.

## Data Availability

The data that support the findings of this study are available from the corresponding author upon reasonable request.
